# Supplementary material for: Genetic evidence points to distinct paternal settlers of the Faroe Islands and Iceland
Source: Front Genet. 2024 Nov 25;15:1462736. doi: 10.3389/fgene.2024.1462736 (PMC11625576; doi:10.3389/fgene.2024.1462736)
Supplement: Supplementary file 1 [file DataSheet2.pdf]

Early founder effects have determined paternal population structure in the Faroe Islands

Allison E. Mann, Eyðfinn Magnussen, Christopher R. Tillquist

Supplementary Figures

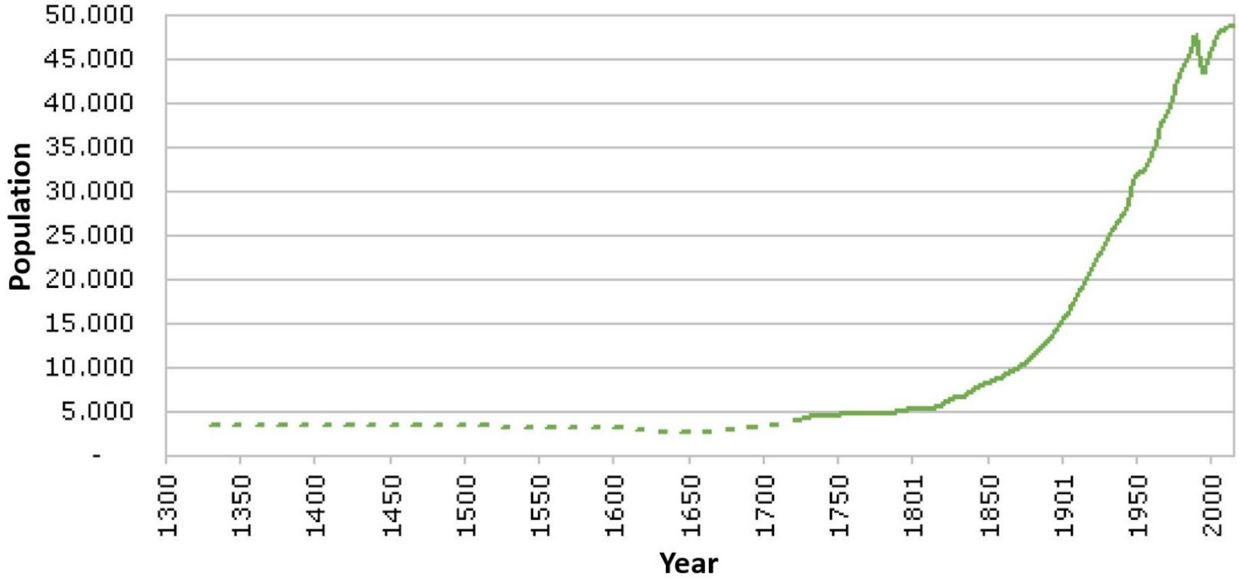

Supplementary Figure 1: Population growth of the Faroese human population during 700 years, from 1330 to 2015. Data from Strøm 2017.

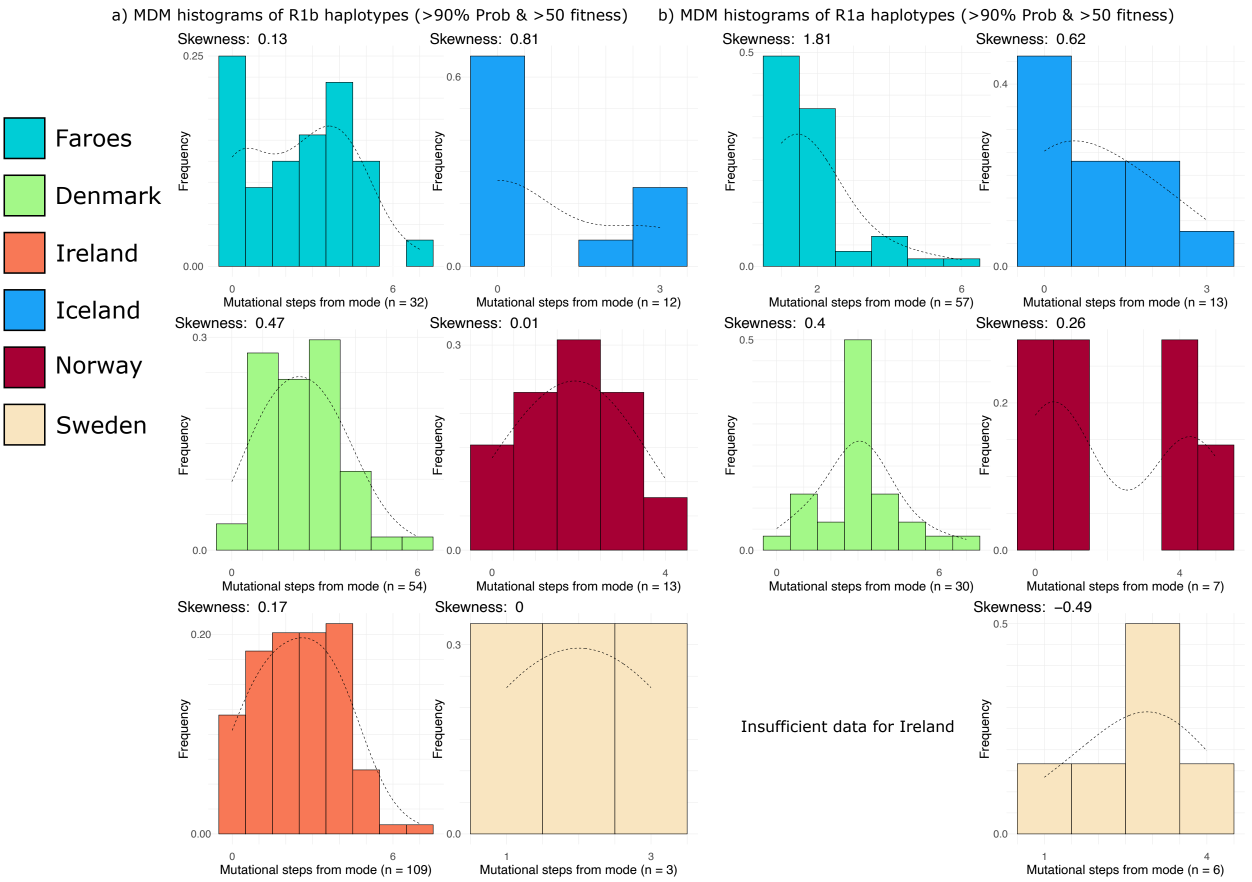

Supplementary Figure 2: MDM histograms using strict haplogroup prediction. a) Population specific MDM histograms of haplotypes assigned to haplogroup R1b that passed strict haplogroup assignment (Probability > 90%, Fitness score > 50). b) Population specific MDM histograms of haplotypes assigned to haplogroup R1a that passed strict haplogroup assignment (Probability > 90%, Fitness score > 50).

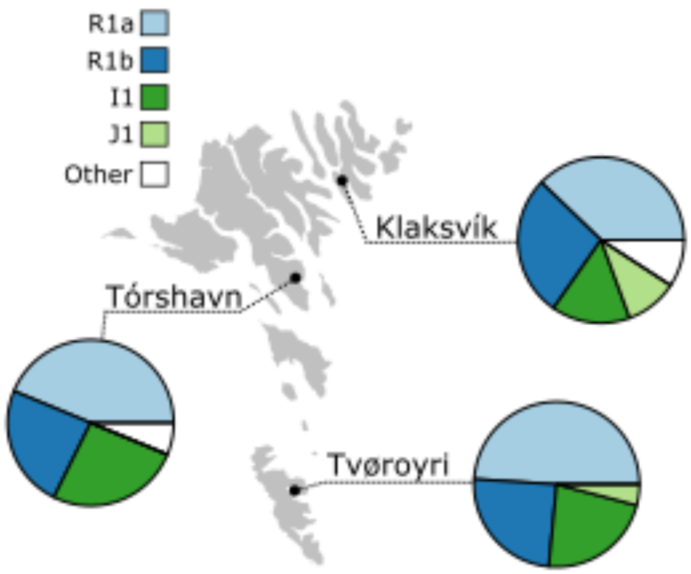

Supplementary Figure 3: Haplogroup frequency by location in the Faroes. Haplogroups with less than 10% total frequency at any location collapsed into “other”.
